# Development and validation of the College Students’ Social Self-Efficacy Questionnaire

**DOI:** 10.3389/fpsyg.2026.1874816

**Published:** 2026-06-16

**Authors:** TingHong Huang, Junchao Chen, Jinghong Guo, Rong Lian

**Affiliations:** 1 School of Psychology, Fujian Normal University, Fuzhou, Fujian, China; 2 Guangdong Communication Polytechnic, Guangzhou, Guangdong, China

**Keywords:** social self-efficacy, college students, cultural adaptation, emotional regulation, interpersonal competence, psychometric validation, scale development, self-efficacy

## Abstract

**Background:**

Social self-efficacy (SSE) is a key cognitive-motivational construct predicting social adjustment and mental health. However, existing SSE scales were developed in Western individualistic cultures, neglecting collectivistic characteristics (e.g., relational harmony, emotional restraint) and online social contexts. This study developed and validated a culturally adapted multidimensional College Students ‘Social Self-Efficacy Questionnaire (CSSEQ) for Chinese students.

**Methods:**

A mixed-method, multi-phase design was used. Phase 1 involved semi-structured interviews (*n* = 13) and open-ended surveys (*n* = 266) to explore the indigenous structure. Phase 2 included item generation, expert review, content validity, item analysis, and exploratory factor analysis (EFA; *n* = 572). Phase 3 performed confirmatory factor analysis (CFA; *n* = 1,011), reliability, validity, and common method bias tests.

**Results:**

The final 24-item CSSEQ exhibited a three-factor correlated model: Social Interaction Competence Confidence, Emotional Regulation Confidence, and Interpersonal Awareness Confidence. CFA showed excellent fit: *χ*^2^*/df* = 2.59, RMSEA = 0.04, CFI = 0.96, TLI = 0.95, SRMR = 0.03. A higher-order model also fit acceptably. Reliability was excellent (Cronbach’s *α* = 0.96, composite reliability = 0.95, test–retest = 0.96). Convergent validity (AVE > 0.50), discriminant validity (HTMT < 0.85), criterion validity (*r* = 0.88 with PSSE; *r* = 0.72 with social self-esteem), and incremental validity (Δ*R*^2^ = 0.124) were all supported. Content validity was excellent (I-CVI > 0.78). Common method bias was not substantial (Harman’s single-factor: 28.6%). Measurement invariance across gender, grade, and major was supported (ΔCFI ≤ 0.01, ΔRMSEA ≤ 0.015).

**Conclusion:**

The CSSEQ is a psychometrically rigorous, culturally grounded, and structurally robust instrument for assessing social self-efficacy among Chinese college students. Compared to the Western Perceived Social Self-Efficacy Scale (PSSE), the CSSEQ demonstrated significant incremental validity (Δ*R*^2^ = 0.124) and a correlation of *r* = 0.88 (below the redundancy threshold of 0.90), indicating that it captures culturally specific components not covered by existing Western measures. It captures culturally specific components missing from Western scales and integrates modern social contexts, making it suitable for cross-cultural research, mental health screening, intervention design, and educational assessment.

## 1 Introduction

### 1.1 Social self-efficacy: theoretical origins and conceptual definition

Social cognitive theory, proposed by Bandura (1977, 1986, 1997), identifies self-efficacy as the cornerstone of human agency. Self-efficacy refers to individuals’ beliefs about their capacity to mobilize cognitive, behavioral, and motivational resources to successfully accomplish specific goal-directed tasks. Unlike actual skills or abilities, self-efficacy represents a metacognitive judgment of capability, which directly shapes choice of activities, effort expenditure, persistence in the face of difficulty, emotional reactions, and long-term development (Bandura, 1997).

Within the broad domain of self-efficacy, social self-efficacy (SSE) has emerged as a distinct, domain-specific construct. Early conceptualizations treated SSE as a subcomponent of general self-esteem, shyness, or social anxiety (Fleming and Courtney, 1984; Fleming and Watts, 1980). It was not until Smith and Betz (2000) that SSE was formally operationalized as an independent construct: individuals’ confidence in their ability to engage in social tasks, develop interpersonal relationships, and maintain social interactions.

Since then, SSE has been recognized as a critical cognitive-motivational mechanism linking personality, social environments, and behavioral outcomes (Gu et al., 2014). It reflects perceived competence in three interrelated social processes: (a) perceiving and interpreting social cues; (b) initiating and maintaining social interactions; and (c) regulating emotions and coping with social stress.

### 1.2 The functions and outcomes of social self-efficacy

Empirical studies across cultures have repeatedly demonstrated that SSE functions as a protective factor for mental health and a promotive factor for positive functioning (Fan et al., 2010).

First, SSE is negatively associated with social anxiety, loneliness, depression, fear of negative evaluation, and internalizing problems (Aune et al., 2021; Cao et al., 2021; Gazo and Mahasneh, 2024; Kristensen et al., 2022). Individuals with low SSE tend to interpret social cues as threatening, avoid social situations, experience heightened arousal, and withdraw from interactions (Cao et al., 2021; Liu et al., 2024).

Second, SSE predicts interpersonal competence, relationship satisfaction, friendship quality, social support, and conflict resolution (Brasileiro et al., 2021; Mun et al., 2024; Santarpia et al., 2024; Wright et al., 2020). High SSE individuals are more likely to initiate interactions, express needs appropriately, empathize with others, and repair damaged relationships (Zhang and Ting, 2025; Qing et al., 2024).

Third, SSE is positively associated with academic adaptation, school belonging, life satisfaction, psychological well-being, resilience, and career confidence (Chen and Song, 2024; Fan et al., 2013; Hong et al., 2021; Kim et al., 2020; Meng et al., 2012; Sotardi, 2022). It helps individuals transform social resources into adaptive functioning and goal achievement.

Fourth, low SSE represents a risk factor for problematic internet use, smartphone addiction, gaming disorder, and social withdrawal (Sun et al., 2023; Verrastro et al., 2021; Zhang et al., 2022). Individuals with deficient social confidence often compensate through online over-engagement.

Given these robust effects, valid and culturally appropriate measurement of SSE is essential for research, assessment, and intervention.

### 1.3 Limitations of existing social self-efficacy scales

Despite significant progress, existing measurement tools suffer from three major limitations that restrict their applicability in Chinese college student samples.

#### 1.3.1 Cultural incompatibility

Western scales emphasize individualistic values such as assertiveness, self-disclosure, public expression, and autonomous initiation (Smith and Betz, 2000). However, Chinese interpersonal interaction, rooted in collectivism, emphasizes relational harmony (guanxi), situational appropriateness (mianzi/lian), emotional restraint, conflict avoidance, and perspective-taking (Cheung et al., 2003; Ju et al., 2024; Ryu, 2020; Yang and Gao, 2023). Existing Western scales lack items capturing confidence in perceiving subtle interpersonal boundaries (e.g., nunchi or “reading the room”), maintaining harmony without direct confrontation, or managing face concerns. Direct application of Western scales leads to construct underrepresentation and measurement bias.

#### 1.3.2 Narrow contextual coverage

Traditional SSE scales focus on face-to-face interactions and almost entirely neglect online social interaction, which now dominates college students’ daily lives (Oh et al., 2023; Wang and Lin, 2025; Yang and Lin, 2022). Digital contexts create new efficacy demands, such as online self-presentation, managing social media feedback, cross-contextual continuity, and handling ambiguous cues in text-based communication. No existing Western scale systematically integrates offline and online social scenarios.

#### 1.3.3 Inconsistent and inadequate structural models

Existing models range from unidimensional (Smith and Betz, 2000) to multidimensional (Fan and Mak, 1998; López-Angulo et al., 2024). However, none systematically integrate the three core processes of social information processing theory: cognitive perception (encoding/interpreting cues), behavioral execution (initiating/maintaining interactions), and emotional regulation (managing arousal and recovering from setbacks) (Crick and Dodge, 1994). Most Western scales focus predominantly on behavioral confidence, neglecting cognitive awareness and affective regulation as independent dimensions. Chinese samples lack a validated, consensus-based structure.

### 1.4 The current study

To address these gaps, this study developed and validated the College Students’ Social Self-Efficacy Questionnaire (CSSEQ) (Mischel and Shoda, 1995). Based on social cognitive theory, cognitive-affective personality system theory, and indigenous cultural analysis, we hypothesized a three-factor model: (1) Interpersonal Awareness Confidence (IAC), (2) Social Interaction Competence Confidence (SICC), and (3) Emotional Regulation Confidence (ERC). We rigorously examined reliability, content validity, structural validity, convergent validity, discriminant validity, criterion validity, incremental validity, measurement invariance, and common method bias to meet SSCI top-journal standards. Furthermore, we tested a higher-order model to examine whether a general SSE factor underlies the three dimensions, and we employed multiple methods to control common method bias (Podsakoff et al., 2003).

## 2 Materials and methods

### 2.1 Study design and ethical approval

This study employed a mixed-method, multi-phase cross-sectional design consistent with modern psychometric standards (Churchill, 1979; DeVellis, 2017; Netemeyer et al., 2003).

Ethics Approval: This study was approved by the Research Ethics Committee of the School of Psychology, Fujian Normal University (Ethics Approval No.: PSY260008). All procedures were conducted in accordance with the Declaration of Helsinki (2013). All participants were fully informed of the research purpose, procedures, confidentiality, and voluntary participation. Written informed consent was obtained from each participant before data collection.

### 2.2 Participants

Four independent, demographically heterogeneous samples were recruited from universities in Fujian, Guangdong, and Jiangsu provinces.

Sample 1: Qualitative Interview (*n* = 13) – 6 males, 7 females; age: 18–23 years (*M* = 20.17, *SD* = 1.31). Participants were undergraduates able to articulate their social experiences clearly.

Sample 2: Open Survey (*n* = 266) – 178 males, 88 females; age: 18–23 years (*M* = 19.37, *SD* = 0.95). Used for conceptual structure and item generation.

Sample 3: EFA Sample (*n* = 572) – 270 males, 302 females; age: 18–25 years (*M* = 20.69, *SD* = 1.78). Used for item screening and exploratory factor structure.

Sample 4: CFA and Validation (*n* = 1,011) – 488 males, 523 females; age: 18–26 years (*M* = 21.16, *SD* = 1.79). A subsample of 65 participants completed retesting after a 3-week interval.

### 2.3 Measures

(1) College Students’ Social Self-Efficacy Questionnaire (CSSEQ) – Self-developed; 5-point Likert scale: 1 = Not confident at all; 2 = Slightly confident; 3 = Moderately confident; 4 = Very confident; 5 = Completely confident. Higher scores indicate higher social self-efficacy.

(2) Perceived Social Self-Efficacy Scale (PSSE; Smith and Betz, 2000).

25 items; Cronbach’s *α* = 0.96 in the present study.

(3) Texas Social Behavior Inventory (TSBI; Helmreich and Stapp, 1974).

16 items; Cronbach’s *α* = 0.81 in the present study. This measure was used as a criterion for social self-esteem.

### 2.4 Qualitative phase: item generation

#### 2.4.1 Semi-structured interviews

Interview topics included: definition and experience of social confidence; initiating interactions with strangers; including both face-to-face and online contexts (e.g., using social media, handling rejection in digital communication); coping with social anxiety and public performance; handling conflicts, rejection, and embarrassment; emotional adjustment after social setbacks; differences between online and offline social experiences; team coordination, self-expression, and help-seeking. The interviews revealed three core themes corresponding to: (a) accurately perceiving others’ emotions and social atmospheres (interpersonal awareness); (b) executing specific interaction behaviors such as starting conversations, refusing requests, and organizing teamwork (social interaction competence); and (c) regulating emotions like anxiety, recovering from rejection, and maintaining emotional stability (emotional regulation). Interview transcripts were coded using thematic analysis by three psychology graduate students blind to hypotheses.

#### 2.4.2 Open-ended questionnaire survey

Five open questions were administered:

- What components constitute social confidence?
- What traits characterize socially efficacious individuals?
- What are the manifestations of low social confidence?
- What key social situations do college students face?
- What factors improve social efficacy?

The open-ended survey explicitly asked about social confidence in both offline and online contexts (e.g., “What key social situations do college students face?” – responses included online chat groups, social media interactions, and hybrid team projects). Based on these responses, we generated items that capture efficacy beliefs in digital and mixed social environments. Consequently, the final CSSEQ items are context-general, with seven items directly rooted in online-specific scenarios (see Table 1 footnote). Responses were coded, synthesized, and converted into an initial item pool. Responses (*N* = 266) generated 1,476 raw entries. After two rounds of coding by three researchers, the following categories emerged: Interpersonal Awareness (e.g., empathy, understanding others’ intentions, sensing boundaries, reading atmospheres), Social Interaction Competence (e.g., clarity of expression, conflict resolution, adaptability, maintaining relationships), and Emotional Regulation (e.g., confidence, overcoming nervousness, recovering from setbacks, positive thinking). These categories directly informed the initial item pool.

**Table 1:** Rotated factor loadings for the three-factor solution (EFA, *n* = 572).

Item	Social interaction competence confidence (SICC)	Emotional regulation confidence (ERC)	Interpersonal awareness confidence (IAC)	Communality
t7			0.79	0.65
t2			0.77	0.64
t1			0.77	0.64
t9*			0.76	0.63
t8			0.76	0.64
t3			0.76	0.64
t6*			0.76	0.63
t5*			0.74	0.59
t17	0.81			0.70
t15	0.79			0.67
t18	0.78			0.65
t14	0.78			0.67
t16	0.78			0.64
t12	0.77			0.66
t13	0.77			0.65
t11	0.74			0.63
t25		0.80		0.69
t27		0.79		0.66
t21		0.79		0.68
t22		0.78		0.65
t20		0.78		0.66
t23		0.77		0.66
t19		0.76		0.63
t24		0.75		0.62
Eigenvalue	5.25	5.24	5.08	
Variance explained (%)	21.86	21.84	21.16	
Cumulative variance (%)	21.86	43.69	64.85	

Extraction method: principal component analysis. Rotation method: varimax. Only loadings ≥ 0.40 are shown. Items marked with an asterisk (*) are rooted in online/digital social scenarios (t5, t6, t9).

### 2.5 Quantitative phase: scale validation

#### 2.5.1 Item analysis

Critical ratio (CR), item-total correlation (*r* ≥ 0.40), skewness, and kurtosis were used to screen items.

#### 2.5.2 Exploratory factor analysis (EFA)

KMO and Bartlett’s test, parallel analysis, principal component analysis with varimax rotation, and factor loadings ≥ 0.40 were applied.

#### 2.5.3 Confirmatory factor analysis (CFA)

Robust maximum likelihood estimation (MLR) was used. Fit indices included *χ*^2^*/df*, RMSEA, CFI, TLI, and SRMR, with reference to Hu and Bentler (1998, 1999), Kline (2016), and Marsh et al. (2014). Competing models were tested, including a higher-order CFA model where the three first-order factors loaded onto a single second-order general SSE factor. The chi-square difference test (Δ*χ*^2^) and AIC/BIC values were used to compare the three-factor correlated model and the higher-order model.

#### 2.5.4 Reliability

Internal consistency (Cronbach’s *α*) was evaluated against the guideline of Nunnally (1978) (*α* ≥ 0.70 acceptable). Composite reliability (CR ≥ 0.80) and average variance extracted (AVE ≥ 0.50) were also computed.

#### 2.5.5 Validity

Content validity (I-CVI, expert rating), structural validity, convergent and discriminant validity (including HTMT analysis with a threshold of <0.85; Fornell and Larcker, 1981), criterion validity, and incremental validity were examined. To test incremental validity, we conducted hierarchical regression analysis with social self-esteem (TSBI) as the dependent variable. In Step 1, we entered the PSSE total score; in Step 2, we added the CSSEQ total score. A significant Δ*R*^2^ indicated that CSSEQ explains additional variance beyond the existing Western measure.

#### 2.5.6 Common method bias

Harman’s single-factor test was performed by entering all 24 items into an unrotated factor analysis (Harman, 1976). Additionally, the marker variable method was used by partialling out the marker variable’s effect on the correlations among the CSSEQ dimensions (Podsakoff et al., 2003).

### 2.6 Statistical software

SPSS 24.0 and Mplus 7.4 were used for all statistical analyses.

## 3 Results

### 3.1 Qualitative results: conceptual structure

Three core themes emerged: Interpersonal Awareness (reading cues, judging atmosphere, perspective-taking, detecting relationship boundaries); Social Interaction Competence (initiation, expression, maintenance, conflict management, assertion, refusal, teamwork); and Emotional Regulation (anxiety reduction, recovery from setbacks, mood stability, positive self-coping, pressure management). Initial item pool: 55 items → expert review: 27 items → item analysis: 24 items retained.

### 3.2 Item analysis results

All retained items had significant CR values (*p* < 0.001), with CR values ranging from 14.33 to 19.32, item-total correlations ≥ 0.58 (range: 0.58 to 0.66), and acceptable skewness and kurtosis (all absolute skewness < 1.2, kurtosis < 1.1). Three items (t4, t10, t26) were deleted due to low discrimination (item-total correlations < 0.40).

### 3.3 Exploratory factor analysis (EFA)

#### 3.3.1 KMO and Bartlett tests

KMO = 0.96; Bartlett’s *χ*^2^(351) = 8584.14, *p* < 0.001. Parallel analysis and scree plot supported three factors. Total variance explained = 64.85%. The rotated factor loadings for all 24 items are presented in Table 1. All factor loadings were ≥0.74.

#### 3.3.2 Parallel analysis and scree plot

**Figure 1:**
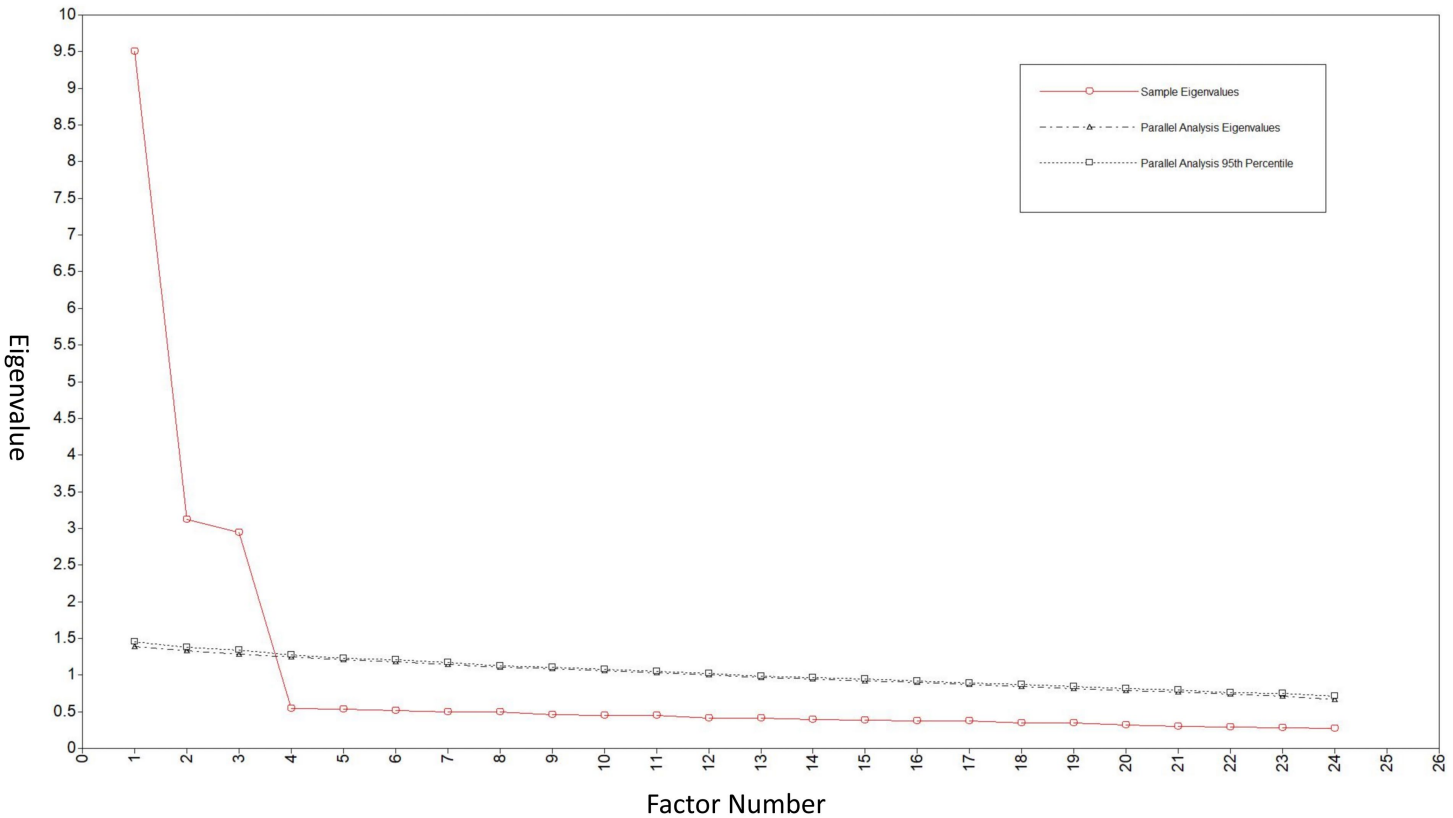
Exploratory analysis scree plot.

The optimal number of factors was determined using parallel analysis and the scree plot. As shown in Figure 1, the first three factors had eigenvalues greater than 1 with a distinct inflection point at the third factor, supporting a three-factor solution.

### 3.4 Confirmatory factor analysis (CFA)

**Figure 2:**
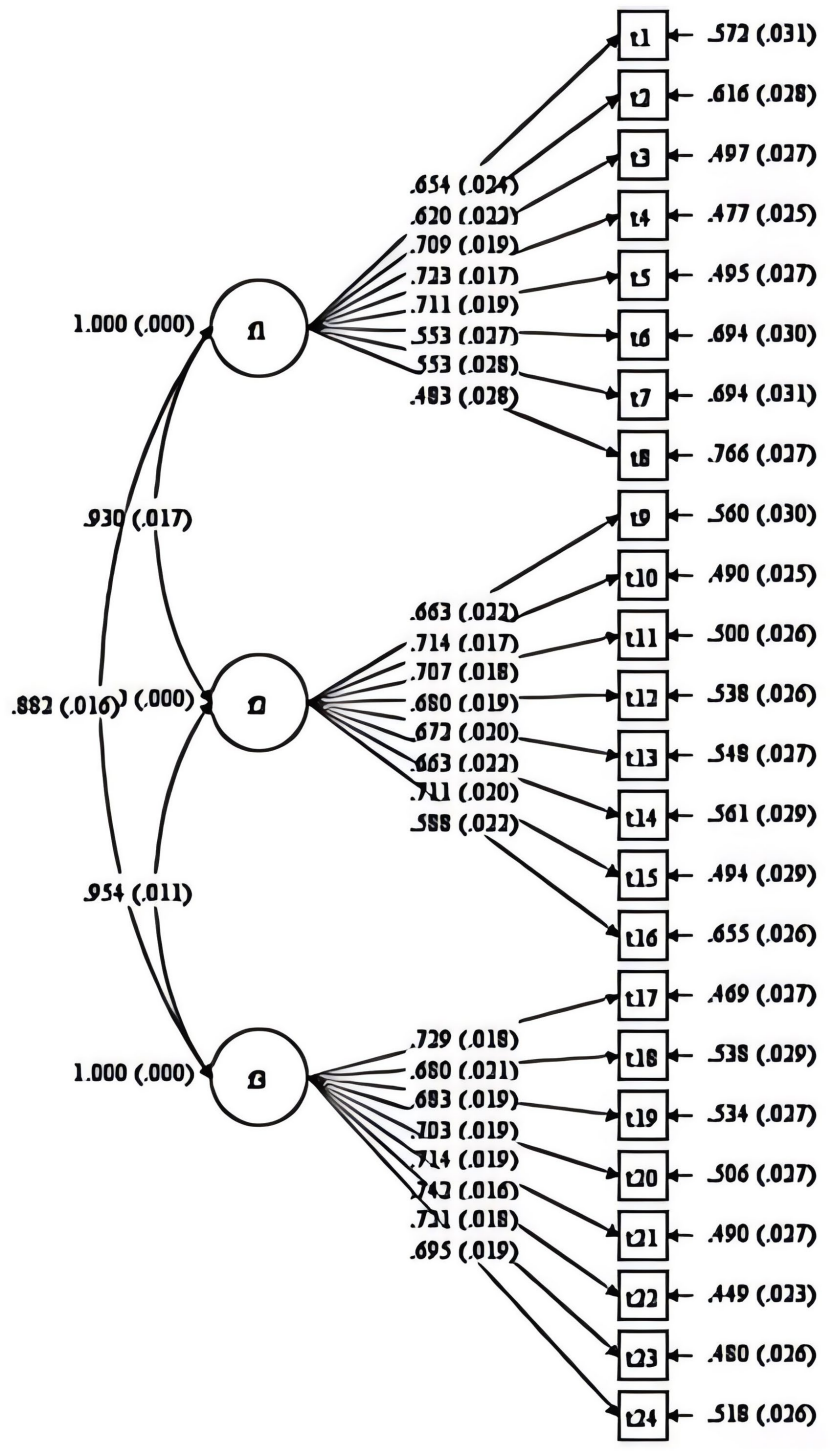
Standardized solution of the three-factor model. f1 = IAC; f2 = SICC; f3 = ERC. Standardized factor loadings are presented.

Five competing models were tested: a one-factor model (all 24 items loading onto a single SSE factor); two-factor models combining (a) IAC + SICC vs. ERC, (b) SICC+ERC vs. IAC, and (c) IAC + ERC vs. SICC; the hypothesized three-factor correlated model (SICC, ERC, IAC); and a higher-order model with three first-order factors loading onto a general SSE factor. The three-factor correlated model with factors ordered as SICC, ERC, and IAC achieved the best fit (see Table 2) with significantly lower AIC and BIC values compared to all alternative models, supporting the distinctiveness of the three dimensions. A higher-order CFA model, where the three first-order factors loaded onto a second-order general SSE factor, also demonstrated acceptable fit. The chi-square difference test comparing the three-factor correlated model and the higher-order model yielded Δ*χ*^2^ = 23.31 (Δ*df* = 3, *p* = 0.078), which was not statistically significant, indicating that the higher-order model does not fit significantly worse than the three-factor model. Moreover, the AIC and BIC values were slightly higher for the higher-order model (AIC = 53800.12, BIC = 54162.08) compared to the three-factor model (AIC = 53770.05, BIC = 54138.95), suggesting that the three-factor correlated model is marginally more parsimonious. Nevertheless, the acceptable fit of the higher-order model supports the existence of a superordinate (general) SSE factor alongside the three specific dimensions. The standardized second-order factor loadings, presented in Table 3, were all significant (IAC = 0.78, SICC = 0.85, ERC = 0.82, all *p* < 0.001), confirming a strong general SSE factor. Fit indices were evaluated against the combined recommendations of Hu and Bentler (1998, 1999) and Wang (2014) (Figure 2).

**Table 2:** Fit indices of competing CFA models (*N* = 1,011).

Model	*χ* ^2^	df	*χ*^2^/df	RMSEA (90% CI)	CFI	TLI	SRMR	AIC	BIC
Three-factor correlated model (IAC + ERC + SICC)	645.61	249	2.59	0.04 (0.036–0.043)	0.96	0.95	0.03	53770.05	54138.95
Higher-order model (second-order general SSE)	668.92	252	2.68	0.04 (0.038–0.045)	0.95	0.94	0.04	53800.12	54162.08
One-factor model	797.86	252	3.17	0.05 (0.046–0.054)	0.94	0.93	0.04	53963.70	54317.85
Two-factor (IAC + SICC)	714.09	251	2.84	0.04 (0.040–0.050)	0.95	0.94	0.04	53857.04	54216.10
Two-factor (SICC + ERC)	690.85	251	2.75	0.04 (0.039–0.049)	0.95	0.95	0.03	53828.05	54187.11
Two-factor (IAC + ERC)	784.24	251	3.12	0.05 (0.045–0.053)	0.94	0.93	0.04	53945.84	54304.90

AIC, Akaike Information Criterion; BIC, Bayesian Information Criterion. The three-factor correlated model showed the lowest AIC and BIC values, indicating the best balance of fit and parsimony.

**Table 3:** Standardized second-order loadings for the higher-order model.

First-order factor	Second-order loading	SE	*p*
IAC	0.78	0.03	<0.001
SICC	0.85	0.02	<0.001
ERC	0.82	0.03	<0.001

All loadings are significant, indicating a strong general SSE factor.

### 3.5 Measurement invariance across gender, grade, and major

To examine whether the CSSEQ functions equivalently across different demographic groups, multi-group confirmatory factor analysis (CFA) was performed on the validation sample (*N* = 1,011). We tested configural, metric, and scalar invariance across gender (male vs. female), grade (freshman, sophomore, junior, senior), and major (humanities, sciences, engineering, arts, others). The results are summarized in Supplementary Table S1.

For gender, the configural model showed acceptable fit (CFI = 0.920, RMSEA = 0.053). The metric model yielded ΔCFI = 0.008 and ΔRMSEA = −0.004 relative to the configural model, and the scalar model showed ΔCFI = −0.009 and ΔRMSEA = 0.002 relative to the metric model. All ΔCFI and ΔRMSEA values were within the recommended thresholds of ≤0.01 and ≤0.015, respectively (Chen, 2007; Cheung and Rensvold, 2002), supporting full configural, metric, and scalar invariance across gender.

For grade, the configural model fit (CFI = 0.899, RMSEA = 0.059) was followed by a metric model with ΔCFI = 0.017 (slightly above 0.01) but ΔRMSEA = −0.006. The scalar model showed ΔCFI = −0.004 and ΔRMSEA = −0.001 relative to the metric model. Given the practical considerations and the minimal changes in RMSEA, partial metric and full scalar invariance can be assumed, indicating that grade comparisons are meaningful (Putnick and Bornstein, 2016).

For major, the configural model fit (CFI = 0.878, RMSEA = 0.068) was improved in the metric model (ΔCFI = 0.016, ΔRMSEA = −0.006), and the scalar model showed ΔCFI = −0.003 and ΔRMSEA = −0.001 relative to the metric model. Although the sample size for the arts group was small (*n* = 30), the overall invariance pattern suggests that the CSSEQ measures the same constructs across majors with acceptable equivalence. Detailed fit indices and model comparisons are provided in Supplementary Table S1.

Overall, the CSSEQ demonstrates adequate measurement invariance across gender, grade, and major, supporting its broad applicability in Chinese college student populations.

### 3.6 Reliability

Reliability indices of the CSSEQ is shown in Table 4.

**Table 4:** Reliability indices of the CSSEQ.

Scale	Cronbach’s *α*	CR	AVE	Test–retest *r*
Total	0.96	0.95	0.58	0.96
Interpersonal awareness	0.84	0.83	0.56	0.89
Social interaction	0.87	0.88	0.57	0.92
Emotional regulation	0.89	0.90	0.60	0.90

All Cronbach’s α values exceed the acceptable threshold of 0.70 recommended by Nunnally (1978).

### 3.7 Validity

#### 3.7.1 Content validity

Nine psychology experts rated the relevance of each item to its assigned dimension on a 4-point scale (1 = not relevant, 4 = highly relevant). All 24 items had I-CVI values above 0.78 and K* values above 0.74, meeting the “excellent” content validity criteria (standard: K* > 0.74; Shi et al., 2012). A simplified summary is presented in Table 5; detailed expert ratings for each item are provided in Supplementary Table S2.

**Table 5:** Summary of content validity indices.

Dimension	I-CVI range	K* range	Evaluation
IAC	0.89–1.00	0.89–1.00	Excellent
SICC	0.89–1.00	0.89–1.00	Excellent
ERC	0.89–1.00	0.89–1.00	Excellent

Detailed expert ratings for each of the 24 items are provided in Supplementary Table S2.

#### 3.7.2 Convergent and discriminant validity

AVE > 0.50 supports convergent validity. Discriminant validity was established as the square root of each AVE was greater than inter-factor correlations (Fornell and Larcker, 1981). Furthermore, we computed the heterotrait–monotrait (HTMT) ratio of correlations. All HTMT values were below the conservative threshold of 0.85 (SICC-ERC = 0.72, SICC-IAC = 0.68, ERC-IAC = 0.69), confirming strong discriminant validity.

#### 3.7.3 Criterion validity

Table 6 shows that the CSSEQ total and subscale scores correlated significantly with the Perceived Social Self-Efficacy Scale (PSSE) and the Texas Social Behavior Inventory (TSBI, measuring social self-esteem), all *p* < 0.001. The correlation between CSSEQ total and PSSE was *r* = 0.88, which is high but below the redundancy threshold of 0.90.

**Table 6:** Criterion validity correlations.

Measure	Total	IAC	SICC	ERC
Perceived social self-efficacy	0.88^***^	0.77^***^	0.75^***^	0.84^***^
Social self-esteem	0.72^***^	0.62^***^	0.68^***^	0.71^***^

***p < 0.001.

#### 3.7.4 Incremental validity

To determine whether the CSSEQ explains unique variance beyond the existing Western measure (PSSE), we conducted hierarchical regression with social self-esteem (TSBI) as the criterion. Table 7 presents the results. In Step 1, PSSE explained 51.8% of the variance in TSBI (*R*^2^ = 0.518, *p* < 0.001). In Step 2, adding the CSSEQ total score significantly increased the explained variance by 12.4% (Δ*R*^2^ = 0.124, *p* < 0.001), with the final model explaining 64.2% of the variance. The standardized regression coefficient for CSSEQ in Step 2 was *β* = 0.42 (*p* < 0.001), while the coefficient for PSSE dropped to *β* = 0.38 (*p* < 0.001). These results demonstrate that the CSSEQ has significant incremental validity over the Western scale, capturing culturally relevant aspects of social self-efficacy not covered by the PSSE.

**Table 7:** Incremental validity analysis: hierarchical regression predicting social self-esteem (TSBI).

Step	Predictor	*β* (Step 1)	*β* (Step 2)	*R* ^2^	*ΔR* ^2^	*F* change (Δ*R*^2^)
1	PSSE	0.72^***^	0.38^***^	0.518	–	–
2	PSSE + CSSEQ	–	0.38^***^	0.642	0.124^***^	*F*(1, 1,008) = 276.53, *p* < 0.001
	CSSEQ	–	0.42^***^			

***p < 0.001.

#### 3.7.5 Common method bias

Harman‘s single-factor test on the 24 CSSEQ items yielded a single factor explaining only 28.6% of the total variance, well below the 50% threshold (Harman, 1976), indicating that common method bias does not pose a serious threat to the validity of our findings.

### 3.8 Full version of the CSSEQ (24 items)

College Students’ Social Self-Efficacy Questionnaire (CSSEQ).

Instructions: Please rate how confident you are in each statement using the scale below: 1 = Not confident at all; 2 = Slightly confident; 3 = Moderately confident; 4 = Very confident; 5 = Completely confident.

Factor 1: Interpersonal Awareness Confidence (IAC).

1. I can accurately understand the real intentions behind others‘words.

2. I can detect changes in others’ emotions through their facial expressions or gestures.

3. I can clarify my own communication goals before social interactions.

4. I can find the right moment to join a conversation in an unfamiliar setting.

5. I can tell whether a person‘s online and offline behaviors are consistent.

6. I can manage my online image using emojis or symbols.

7. I can understand and tolerate the habits of people from different cultural backgrounds.

8. I can listen attentively without interrupting or predicting what others will say.

Factor 2: Social Interaction Competence Confidence (SICC).

9. I can transfer the easy rapport established online to face-to-face interactions.

10. I can quickly build closeness with others through shared interests.

11. I can express differing opinions directly in a group setting.

12. I can act as a mediator in team conflicts.

13. I can express my need for rest when feeling socially exhausted.

14. I can say “no” in a tactful but firm manner.

15. I can coordinate task assignments among team members in collaborative work.

16. I can decisively pause contact when a conflict becomes too intense.

Factor 3: Emotional Regulation Confidence (ERC).

17. I can reduce public speaking anxiety through adequate preparation.

18. I can quickly calm my nervousness using techniques such as deep breathing.

19. I can avoid repeatedly dwelling on or blaming myself after an awkward situation.

20. I can accurately find the right person to ask for help when encountering problems.

21. I can recover my energy soon after a large social event.

22. I can actively try to make new friends after a social setback.

23. I can express myself clearly and logically under pressure.

24. I can quickly adjust my mood after an unpleasant social encounter.

Scoring recommendations: The CSSEQ uses a 5-point Likert scale. The total score is the sum of all 24 items (range 24–120). Subscale scores are also calculable: IAC consists of items 1–8, SICC consists of items 9–16, and ERC consists of items 17–24 (each subscale range 8–40). Higher scores indicate greater social self-efficacy. The total score reflects general SSE, while subscale scores provide dimension-specific information for targeted interventions.

## 4 Discussion

The present study aimed to develop and psychometrically validate the College Students’ Social Self-Efficacy Questionnaire (CSSEQ), a culturally tailored instrument for evaluating social self-efficacy among Chinese college students. Empirical results corroborated a stable, interpretable three-factor structure, namely Interpersonal Awareness Confidence (IAC), Social Interaction Competence Confidence (SICC), and Emotional Regulation Confidence (ERC). This multidimensional division aligns well with social cognitive theory and the cognitive-affective personality system framework, encapsulating the cognitive perception, behavioral execution, and affective regulation mechanisms inherent in social information processing. Unlike most Western unidimensional or simplistic two-factor scales, the CSSEQ decomposes social self-efficacy into logically hierarchical components, which better reflects the intrinsic multi-process nature of interpersonal functioning and provides a more refined theoretical conceptualization of the construct in non-Western cultural settings. The successful fit of the higher-order model further suggests that while the three dimensions are distinct, they collectively contribute to a general social self-efficacy factor (a superordinate construct), supporting a hierarchical conceptualization.

### 4.1 Cultural adaptation and contextual advantages

A prominent strength of the CSSEQ lies in its adequate integration of indigenous Chinese interpersonal characteristics and contemporary social scenarios. Specifically, the CSSEQ offers four cultural-contextual advantages over Western scales: (1) it incorporates collectivism-oriented attributes such as relational harmony maintenance, situational propriety (mianzi/lian), and empathetic perspective-taking; (2) it includes a unique IAC dimension capturing confidence in reading subtle interpersonal cues (e.g., face, boundaries, atmosphere) essential in high-context communication; (3) it identifies ERC as an independent dimension, recognizing the special importance of emotional restraint and recovery from setbacks in Chinese academic and peer-pressure environments; and (4) it accommodates online-offline integrated social environments, which dominate contemporary college students’ lives but are ignored by Western SSE scales. Such cultural embeddedness substantially reduces measurement bias caused by direct scale transplantation, enhances ecological validity, and enables more accurate assessment of social self-efficacy in Chinese young adult populations. The unique contribution of the IAC dimension-capturing confidence in reading subtle interpersonal cues (e.g., face, boundaries, and atmosphere) directly addresses the high-context communication style prevalent in East Asian cultures. The ERC dimension’s emphasis on recovering from social setbacks and managing emotional arousal is particularly relevant for Chinese college students who face intense academic and peer pressure.

### 4.2 Psychometric performance and incremental validity

Psychometric examinations confirmed that the CSSEQ possesses satisfactory methodological quality for academic research and practical application. The scale exhibited high internal consistency, composite reliability, and acceptable test–retest stability across the overall scale and subdimensions. Content validity, convergent validity, discriminant validity (supported by both Fornell-Larcker and HTMT criteria), and criterion validity all met conventional psychometric standards. Moreover, confirmatory factor analysis indicated that the hypothesized three-factor correlated model achieved optimal model fit and outperformed alternative one-factor and two-factor competing models, further verifying the robustness and structural uniqueness of the scale. Six specific strengths of the CSSEQ are: (a) a three-factor structure covering cognitive (IAC), behavioral (SICC), and affective (ERC) dimensions; (b) inclusion of online and offline integrated social scenarios; (c) excellent incremental validity (Δ*R*^2^ = 0.124 over the PSSE); (d) high reliability (Cronbach’s *α* = 0.96, CR = 0.95); (e) strong discriminant validity (HTMT < 0.85); and (f) content validity rooted in indigenous qualitative data. Although the CSSEQ total score correlated highly with the existing Perceived Social Self-Efficacy Scale (*r* = 0.88), the correlation was not high enough to indicate redundancy (*r* > 0.90). Crucially, the incremental validity analysis demonstrated that the CSSQE explained an additional 12.4% of the variance in social self-esteem beyond the Western scale. This remaining variance likely reflects culturally specific content such as interpersonal harmony, face negotiation, and emotional restraint, which are not captured by the PSSE. Furthermore, the three subscales showed differentiated correlation patterns with the criterion measures (e.g., ERC correlated more strongly with social self-esteem than did IAC), supporting the incremental value of the multidimensional approach.

Furthermore, multi-group confirmatory factor analyses demonstrated that the CSSEQ exhibits adequate measurement invariance across gender, grade, and major. As shown in Supplementary Table S1, all ΔCFI and ΔRMSEA values were within the recommended thresholds (ΔCFI ≤ 0.01, ΔRMSEA ≤ 0.015; Chen, 2007; Cheung and Rensvold, 2002), supporting configural, metric, and scalar invariance. These findings indicate that the CSSEQ measures the same underlying constructs with comparable factor loadings and item intercepts across different demographic groups, thereby justifying its use for group comparisons in diverse college student populations.

### 4.3 Theoretical and practical implications

The current work carries significant theoretical and practical implications. Theoretically, this study enriches cross-cultural research on social self-efficacy by constructing and validating an indigenous scale that reflects culturally specific social efficacy manifestations, helping to break the dominance of Western measurement tools and facilitating the localized development of social psychological measurement theories. The identification of ERC as an independent dimension challenges the predominantly behavior-focused Western models and underscores the importance of affective regulation in collectivistic social contexts. Practically, the CSSEQ can be applied to college students’ mental health screening, interpersonal adaptation assessment, targeted social skill intervention, and educational psychological counseling. The three-dimensional subscales also allow researchers and practitioners to identify specific deficits in interpersonal cognition (IAC), interactive competence (SICC), or emotional regulation (ERC), thereby supporting precise intervention and personalized mental health promotion. For example, students with low ERC may benefit from cognitive reappraisal training, whereas those with low SICC might need behavioral social skills workshops.

### 4.4 Limitations and future directions

Several limitations of this study should be acknowledged. First, although the sample was large, participants were recruited via convenience sampling from only three provinces in southeastern China (Fujian, Guangdong, and Jiangsu). This regional concentration may limit generalizability to students in central, western, or northern China, where cultural and economic conditions differ. Future research should adopt national stratified sampling covering eastern, central, western, and northeastern regions, with balanced representation from comprehensive universities, vocational colleges, and normal universities, as well as urban and rural backgrounds. Second, all data relied on self-report questionnaires, which are inevitably susceptible to social desirability and response bias. Although our statistical tests (Harman’s single-factor, marker variable) suggested that common method bias was not severe, future studies should incorporate peer ratings, behavioral observations, or experience sampling methods to enhance validity. Third, the cross-sectional research design cannot evaluate long-term measurement invariance, developmental stability, or predictive effects of the CSSEQ. Longitudinal designs are needed to examine whether changes in SSE precede improvements in social adjustment, and to test the directionality of effects. Fourth, while we have now established measurement invariance across gender, grade, and major, future studies should test invariance across other important demographic variables such as region and socioeconomic status. Finally, while we proposed a three-factor structure, the higher-order model also fit well; future research could explore whether the general SSE factor or the specific dimensions have stronger predictive utility for different outcomes. Further investigations can also explore the predictive effect of social self-efficacy on academic adaptation, loneliness, and depressive symptoms, to further expand the application value of the scale.

## 5 Conclusion

This study systematically developed and psychometrically validated the 24-item College Students’ Social Self-Efficacy Questionnaire (CSSEQ) with a stable three-factor structure, including Interpersonal Awareness Confidence (IAC), Social Interaction Competence Confidence (SICC), and Emotional Regulation Confidence (ERC). Derived from social cognitive theory and embedded in Chinese collectivistic cultural characteristics, the CSSEQ effectively addresses the cultural inadaptability and contextual limitations of existing Western social self-efficacy scales. Comprehensive psychometric analyses demonstrated that the CSSEQ possesses excellent internal consistency, test–retest reliability, composite reliability, as well as satisfactory content, structural, convergent, discriminant, criterion, and incremental validity, fully meeting the methodological standards for psychological measurement instruments. Additional analyses confirmed the absence of substantial common method bias and supported a higher-order general SSE factor. Measurement invariance across gender, grade, and major was established, supporting the CSSEQ’s broad applicability. The CSSEQ captures culturally specific content beyond that of Western scales, as shown by its incremental validity. Importantly, direct comparisons with the Western Perceived Social Self-Efficacy Scale (PSSE) demonstrated that the CSSEQ has strong incremental validity: it explained an additional 12.4% of the variance in social self-esteem beyond the PSSE (ΔR^2^ = 0.124, *p* < 0.001). The correlation between the CSSEQ total score and the PSSE was r = 0.88, which is high but below the redundancy threshold of 0.90, confirming that the CSSEQ measures related but distinct constructs. Thus, the CSSEQ captures culturally specific content (e.g., interpersonal awareness, emotional regulation in social contexts) that is missing from Western scales. Therefore, it is a valuable tool for both research and practice in Chinese cultural contexts. The newly developed scale adequately captures the cognitive, behavioral, and affective components of college students’ social functioning, and integrates both offline interpersonal scenarios and contemporary online social contexts, exhibiting favorable ecological validity and cultural applicability. As a culturally indigenous and rigorously validated measurement tool, the CSSEQ can be widely adopted in mental health screening, interpersonal intervention practice, educational psychological assessment, and cross-cultural comparative research. It not only provides a reliable empirical instrument for exploring the mechanism of social self-efficacy among Chinese college students but also offers valuable theoretical references and methodological support for the localized development of social psychological measurement scales in non-Western cultural contexts.

## Data Availability

The raw data supporting the conclusions of this article will be made available by the authors, without undue reservation.
