# Supplementary material for: Development and validation of the College Students’ Social Self-Efficacy Questionnaire
Source: Front Psychol. 2026 Jun 16;17:1874816. doi: 10.3389/fpsyg.2026.1874816 (PMC13315009; doi:10.3389/fpsyg.2026.1874816)
Supplement: Supplementary file 1 [file Table_1.docx]

Supplementary Table S1 Measurement Invariance Tests Across Gender, Grade, and Major

| **Grouping variable** | **Model** | ***χ²*** | ***df*** | **CFI** | **RMSEA**  **(90% CI)** | **SRMR** | **ΔCFI** | **ΔRMSEA** |
| --- | --- | --- | --- | --- | --- | --- | --- | --- |
| Gender (male, n=488; female, n=523) | Configural | 1210.57 | 504 | 0.92 | 0.053 (0.049–0.056) | 0.27 | – | – |
|  | Metric | 1165.46 | 522 | 0.93 | 0.049 (0.046–0.053) | 0.25 | 0.008 | –0.004 |
|  | Scalar | 1259.30 | 543 | 0.92 | 0.051 (0.047–0.055) | 0.25 | –0.009 | 0.002 |
| Grade (freshman=245, sophomore=255, junior=288, senior=223) | Configural | 1905.26 | 1008 | 0.90 | 0.059 (0.055–0.063) | 0.29 | – | – |
|  | Metric | 1803.31 | 1062 | 0.92 | 0.053 (0.048–0.057) | 0.16 | 0.017 | –0.006 |
|  | Scalar | 1902.01 | 1125 | 0.91 | 0.052 (0.048–0.056) | 0.16 | –0.004 | –0.001 |
| Major (humanities=275, science=385, engineering=220, arts=30, others=101) | Configural | 2438.77 | 1260 | 0.88 | 0.068 (0.064–0.072) | 0.29 | – | – |
|  | Metric | 2356.84 | 1332 | 0.89 | 0.062 (0.058–0.066) | 0.18 | 0.016 | –0.006 |
|  | Scalar | 2470.45 | 1416 | 0.89 | 0.061 (0.057–0.065) | 0.18 | –0.003 | –0.001 |

Note. All models were estimated using robust maximum likelihood (MLR). ΔCFI and ΔRMSEA values represent changes from the less constrained model to the more constrained model (metric vs. configural; scalar vs. metric). Acceptable invariance is indicated by ΔCFI ≤ 0.01 and ΔRMSEA ≤ 0.015 (Chen, 2007; Cheung & Rensvold, 2002). For grade and major, metric invariance showed ΔCFI slightly above 0.01, but the ΔRMSEA remained within limits, and scalar invariance was fully supported, suggesting practical equivalence across groups. The arts group had a small sample size $\text{n}\text{=30}$, which may affect stability; results should be interpreted with caution.
