# Supplementary material for: Development and validation of the College Students’ Social Self-Efficacy Questionnaire
Source: Front Psychol. 2026 Jun 16;17:1874816. doi: 10.3389/fpsyg.2026.1874816 (PMC13315009; doi:10.3389/fpsyg.2026.1874816)
Supplement: Supplementary file 2 [file Table_2.docx]

Supplementary Table S2 **Content Validity of Each Item of the College Students' Social Self-Efficacy Scale**

| **Dimension** | **Item** | **Expert rating** | | | | | | | | | **I-CVI** | **Pc** | **K*** |
| --- | --- | --- | --- | --- | --- | --- | --- | --- | --- | --- | --- | --- | --- |
|  |  | **A** | **B** | **C** | **D** | **E** | **F** | **G** | **H** | **I** |  |  |  |
| **IAC** | t1 | 4 | 4 | 4 | 4 | 4 | 4 | 4 | 4 | 4 | 1.00 | 0.22 | 1 |
|  | t2 | 4 | 4 | 4 | 4 | 4 | 4 | 4 | 4 | 4 | 1.00 | 0.22 | 1 |
|  | t3 | 3 | 4 | 4 | 3 | 4 | 4 | 4 | 4 | 4 | 1.00 | 0.22 | 1 |
|  | t4 | 4 | 4 | 4 | 4 | 3 | 4 | 4 | 4 | 4 | 1.00 | 0.22 | 1 |
|  | t5 | 4 | 4 | 4 | 3 | 3 | 4 | 4 | 4 | 4 | 1.00 | 0.22 | 1 |
|  | t6 | 4 | 4 | 4 | 4 | 4 | 4 | 4 | 4 | 4 | 1.00 | 0.22 | 1 |
|  | t7 | 3 | 4 | 4 | 3 | 3 | 4 | 4 | 4 | 4 | 1.00 | 0.22 | 1 |
|  | t8 | 3 | 4 | 4 | 3 | 4 | 4 | 4 | 4 | 4 | 1.00 | 0.22 | 1 |
|  | t9 | 4 | 4 | 4 | 4 | 4 | 4 | 4 | 4 | 4 | 1.00 | 0.22 | 1 |
| **SICC** | t10 | 4 | 4 | 4 | 4 | 4 | 4 | 4 | 4 | 4 | 1.00 | 0.22 | 1 |
|  | t11 | 4 | 4 | 4 | 3 | 4 | 4 | 4 | 4 | 4 | 1.00 | 0.22 | 1 |
|  | t12 | 4 | 4 | 4 | 4 | 4 | 4 | 4 | 4 | 4 | 1.00 | 0.22 | 1 |
|  | t13 | 4 | 4 | 4 | 4 | 4 | 4 | 4 | 4 | 4 | 1.00 | 0.22 | 1 |
|  | t14 | 4 | 4 | 4 | 3 | 4 | 4 | 4 | 4 | 4 | 1.00 | 0.22 | 1 |
|  | t15 | 4 | 4 | 4 | 3 | 4 | 4 | 4 | 4 | 4 | 1.00 | 0.22 | 1 |
|  | t16 | 4 | 4 | 4 | 4 | 4 | 4 | 4 | 4 | 4 | 1.00 | 0.22 | 1 |
|  | t17 | 4 | 4 | 4 | 4 | 4 | 4 | 4 | 4 | 4 | 1.00 | 0.22 | 1 |
|  | t18 | 3 | 4 | 4 | 3 | 4 | 4 | 4 | 4 | 4 | 1.00 | 0.22 | 1 |
| **ERC** | t19 | 4 | 4 | 4 | 4 | 3 | 4 | 4 | 4 | 4 | 1.00 | 0.22 | 1 |
|  | t20 | 4 | 4 | 4 | 4 | 4 | 2 | 4 | 4 | 4 | 0.89 | 0.02 | 0.89 |
|  | t21 | 4 | 4 | 4 | 3 | 4 | 4 | 4 | 4 | 4 | 1.00 | 0.22 | 1 |
|  | t22 | 4 | 4 | 4 | 3 | 3 | 3 | 4 | 4 | 4 | 1.00 | 0.22 | 1 |
|  | t23 | 3 | 4 | 4 | 4 | 4 | 3 | 4 | 4 | 4 | 1.00 | 0.22 | 1 |
|  | t24 | 4 | 4 | 4 | 4 | 4 | 3 | 4 | 4 | 4 | 1.00 | 0.22 | 1 |
|  | t25 | 4 | 4 | 4 | 3 | 4 | 3 | 4 | 4 | 4 | 1.00 | 0.22 | 1 |
|  | t26 | 4 | 4 | 4 | 4 | 4 | 3 | 4 | 4 | 4 | 1.00 | 0.22 | 1 |
|  | t27 | 4 | 4 | 4 | 4 | 4 | 3 | 4 | 4 | 4 | 1.00 | 0.22 | 1 |

Note: I-CVI = item-level content validity index (proportion of experts rating 3 or 4); Pc = chance agreement; K* = modified kappa. All K* values exceed the excellent threshold of 0.74.
